# The Impact of Insect Flour on Sourdough Fermentation-Fatty Acids, Amino-Acids, Minerals and Volatile Profile

**DOI:** 10.3390/insects13070576

**Published:** 2022-06-24

**Authors:** Beldean (Tătar) Bianca Vasilica, Maria Simona Chiș, Ersilia Alexa, Carmen Pop, Adriana Păucean, Simona Man, Marta Igual, Kovacs Melinda Haydee, Kovacs Emoke Dalma, Sorin Stănilă, Sonia Socaci, Anca Fărcaș, Adina Berbecea, Iuliana Popescu, Sevastița Muste

**Affiliations:** 1 Department of Food Engineering, Faculty of Food Science and Technology, University of Agricultural Sciences and Veterinary Medicine of Cluj-Napoca, 3-5 Manăștur Street, 400372 Cluj-Napoca, Romania; bianca.beldean@usamvcluj.ro (B.B.V.); adriana.paucean@usamvcluj.ro (A.P.); simona.man@usamvcluj.ro (S.M.); sevastita.muste@usamvcluj.ro (S.M.); 2 Department of Food Control, Faculty of Agro-Food Technologies, Banat University of Agricultural Sciences and Veterinary Medicine “King Michael I of Romania”, 30064 Timisoara, Romania; ersiliaalexa@usab-tm.ro; 3 Department of Food Science, Faculty of Food Science and Technology, University of Agricultural Sciences and Veterinary Medicine of Cluj-Napoca, 3-5 Mănăștur Street, 400372 Cluj-Napoca, Romania; carmen-rodica.pop@usamvcluj.ro (C.P.); sonia.socaci@usamvcluj.ro (S.S.); anca.farcas@usamvcluj.ro (A.F.); 4 Food Investigation and Innovation Group, Food Technology Department, Universitat Politècnica de València, Camino de Vera s/n, 46022 Valencia, Spain; marigra@upvnet.upv.es; 5 NCDO-INOE 2000, Research Institute for Analytical Instrumentation, 67 Donath Street, 400293 Cluj-Napoca, Romania; melinda.kovacs@icia.ro (K.M.H.); melindahaydeekovacs@gmail.com (K.E.D.); 6 Department of Technical Sciences and Soil Sciences, University of Agricultural Sciences and Veterinary Medicine Cluj-Napoca, Calea Mănăștur Street, No. 3-5, 400372 Cluj-Napoca, Romania; 7 Department of Soil Sciences, Faculty of Agriculture, Banat University of Agricultural Sciences and Veterinary Medicine “King Michael I of Romania”, 30064 Timisoara, Romania; adinaberbecea@usab-tm.ro (A.B.); iulianapopescu@usab-tm.ro (I.P.)

**Keywords:** *Acheta domesticus*, insect flour, *Lactobacillus plantarum*, fermentation, bioactive compounds

## Abstract

**Simple Summary:**

*Acheta domesticus* (house cricket) flour is one of the most promising and sustainable sources of nutrients. It is rich in protein, minerals, fatty acids, amino acids, and vitamins and it has a low impact on the consumption of natural environmental resources. On the other side, fermentation with lactic acid bacteria represents a technological tool that can improve further the nutritional quality of flours. Therefore, the aim of the present research was to study the adaptability of the *Lactobacillus plantarum* strain on insect flour fermentation. Fatty acids, amino acids, minerals, and aroma volatile compounds were analyzed during 48 h of fermentation. Fermentation improved the nutritional quantity of the bioactive compounds, mainly after 24 h of fermentation, where they reached higher extended values. Overall, our findings indicate that insect flour is able to support the growth and development of the *Lactobacillus plantarum* strain, leading to an enriched insect flour sourdough that could be further used in the manufacturing of new products.

**Abstract:**

*Acheta domesticus* (L.1758) has been recently accepted by the European Union as a novel food, being the third insect that has been approved for human consumption. Nowadays, researchers’ attention is focused on exploiting new protein sustainable sources, and, therefore, insect flour has gained more and more interest. Organic acids, fatty acids, amino acids, aroma volatile compounds, and minerals were analyzed through HPLC-RID (High-performance liquid chromatography), GC-MS (Gas chromatography-mass spectrometry), LC-MS (Liquid chromatography–mass spectrometry), ITEX/GC-MS and AAS (Atomic Absorption Spectrophotometry), respectively. Fermentation of the insect flour with *Lactobacillus plantarum* ATCC 8014 strain (Lp) leads to an increase in organic acids such as lactic, acetic, and oxalic, whilst citric acid decreases its value. SFA (saturated fatty acids) and MUFA (monosaturated fatty acids) groups were positively influenced by Lp fermentation; meanwhile, PUFA (polysaturated fatty acids) decreased during fermentation. A positive trend was observed for amino acids, aroma volatile content, and minerals enhancement during insect sourdough fermentation, mainly at 24 h of fermentation. *Acheta domesticus (A. domesticus)* sourdough fermentation represents a new tool that needs to be further exploited aiming to improve the nutritional qualities of the final products.

## 1. Introduction

Nowadays, due to the researchers increased interest to have quality protein with less impact on the environmental conditions, significant steps have been made forward the valorization of edible insects. Insects, compared to conventional animals are offering superior or similar nutritional qualities with fewer requirements on water, feed, and land [1]. Their chemical composition rich in proteins, essential amino acids, fatty acids, minerals, and vitamins make them an optimum source of nutrition, being consumed by 2.5 billion people worldwide [2] in more than 110 countries [3]. Moreover, with respect to production efficiency in order to obtain 1 kg of edible product, insects consume 2.1 dried feed; meanwhile, animals such as pig, cattle, or poultry need 9.1 kg, 25 kg, and 4.5 kg, respectively, to produce the same quantity of edible product [4].

Furthermore, edible insects could be considered a sustainable source in many regions such as South and Central America, Australia Papua New Guinea, and South-East Asia region [5], being able to deal with global food security problems [3]. It is expected that the consumption of edible insects will increase by 47% between 2019 and 2026, mainly in Europe and North America [1]. In fact, in 2018, the European Food Safety Authority (EFSA) recognized insects as novel foods through the Regulation (EU) 2015/2283, and the Food Agriculture Organization (FAO) recommended including them in Western diets, aiming to cover the nutritional population gaps in proteins and fats [6,7]. Nowadays, insects are used in food manufacturing such as bread, pasta, muffins, extruded snacks, or even as meat analogs [7,8,9,10]. For around 2 billion people worldwide, insect species such as crickets, ants, grasshoppers, locusts, wasps, or bees are considered as food [11]. The global edible insect market is expected to grow in the period 2020 to 2027 at a CAGR (annual growth rate compound) of 28.5%, reaching a final value of 1,398,862.5 tones by 2027 [12].

*A. domesticus* is one of the most commonly consumed insect varieties, having an interesting and promising nutritional profile, with protein and lipid amounts comparable to those of conventional animals such as chicken or even beef [13]. It is considered that the house cricket contains more than 60% protein and might be harvested from lands with minimal requirements and without persistent chemicals. Moreover, 80% of their body is able to produce food; in the meantime, body cattle are able to produce food in a percentage of only 40% [14]. Furthermore, crickets (*A. domesticus)* are considered by the European Food Safety (EFSA) as insects with the biggest food and feed potential [15].

Food fortification is defined as a strategy, used not only in industrialized countries but also in developing ones, that aims to fill the nutritional gap of a common diet. Therefore, recently, due to its high digestibility protein degree, insect flour became the basis for the fortification of numerous food products [16]. Sourdough is defined as a tool used for enhancing the bioactive compounds of the raw materials [17], and insect flour could represent a substrate that might be able to fulfill the requirements of a lactic acid bacteria growth [18]. 

*Lactobacillus plantarum* is a well-known facultative heterofermentative strain mainly due to its good environmental adaptation, surnamed as having a nomadic lifestyle, being identified also in the intestinal insects’ microbiota [19,20].

Therefore, the aim of the present work is to study the adaptability of *Lactobacillus plantarum* ATCC 8014 strain (Lp) on the insect flour substrate, targeting its influence on amino acids, volatile compounds, fatty acids, organic acids, and minerals compounds.

## 2. Materials and Methods

### 2.1. Materials and Reagents

Insect flour was purchased from the market and was manufactured in Thailand (JR Unique company Foods Ltd., Udon Thani, Thailand). *Lactobacillus plantarum* ATCC 8014 strain was achieved from Microbiologics (St. Cloud, MN, USA) and MRS medium was acquired from Merck (Darmstadt, Germany). Organic acids standards (lactic, acetic, fumaric, citric, succinic, oxalic, ascorbic) were bought from Fluka (Saint Louis, MO, USA) and were 99.5% pure. All the reagents were analytical grade.

### 2.2. Proximate Composition, Microbial Starter Culture Preparation, Sourdough Preparation

Protein, ash, moisture, and total lipid were analyzed according to AACC-approved methods: 46-11A, 08-01, 45-15 A, 30-10.01 [21]. The nitrogen conversion factor for protein was ×5.33, according to Boulous et al. [22]. *Lactobacillus plantarum* ATCC 8014 was cultivated in MRS broth for 48 h at a temperature of 37 °C, as previously described in our studies [17,23]. Briefly, the inoculum was obtained from freeze-dried cells suspended in 10 mL MRS broth, incubated under aerobic conditions at a temperature of 37 °C for 48 h, and then sub-cultured into 95 mL MRS and incubated in the same conditions. Afterward, the biomass was centrifuged at 2300× *g* (Eppendorf R 5804 centrifuge, Hamburg, Germany) for 10 min, at a temperature of 4 °C, washed three times with sterile water, and inoculated in the prepared matrix in order to achieve an initial cell account of log 3.2. cfu/mL sourdough. Microorganism concentration was determined with spectrophotometer NanoDrop 1000 (NanoDrop Technologies, Wilmington, DE, USA) based on optical density measurement at 600 nm (OD600). Sourdoughs were prepared by mixing insect flour with tap water and inoculum in a ratio of 100:80:20 to obtain a final dough yield of 200 mL, as previously described by Chiș et al. [24]. Briefly, 100 g of flour was mixed manually with 80 mL of tap water and 20 mL of inoculum. The fermentation was realized for 48 h, at a temperature of 37 °C, and samples were withdrawn at the following times: 0, 4, 8, 12, 24, and 48 h respectively. A control sample without inoculum (spontaneous fermentation) and without previous sterilization was prepared and fermented in the same conditions. Samples were coded as presented in Table 1.

### 2.3. pH, TTA, and Microbial Counts

The pH, total titratable acidity (TTA), and microbial counts were determined according to the methods described by our research group [23,24]. WTW pH-meter (Hanna Instruments, Vöhringen, Germany) was used for pH and TTA analysis. Briefly, 10 g of sourdough was mixed with 90 mL distilled water and the obtained solution was neutralized with NaOH, 0.1 N, till a final pH of 8.3 units was achieved. Phenolphthalein was used as a color indicator and TTA values were expressed as mL NaOH.

With respect to microbial counts, 5 mL of each sample was withdrawn at different hours and mixed with 45 mL sterile sodium chloride (0.85% *w*/*v*). From the obtained solutions, 1 mL was used for serial dilutions and plating in the prepared MRS agar. The plates were incubated under the following conditions: 30 °C, 48 h. A colony counter Colony Star 8500, (Funke-Dr. N.Gerber Labortechnik, Berlin, Germany) was used to count the visible colonies.

### 2.4. Organic Acids and Alcohols

Organic acids and alcohols were analyzed by using HPLC-RID: High-performance liquid chromatography (HPLC-Agilent 1200 series, Santa Clara, CA, USA) coupled with a refractive index detector (Agilent Technologies, Santa Clara, CA, USA), as described by Chiș et al. [17]. Shortly afterward, 1 g of each sample was extracted with 4 mL ultrapure water, mixed using a Heidoph Reax top vortex (Merck, Darmstadt, Germany) for 1 min and sonicated for 30 min using an ultrasonic bath (Elma Schmidbauer, GmbH, Singen, Germany). An Eppendorf 5804 centrifuge (Eppendorf, Hamburg, Germany) was used to centrifugate samples at 2300× *g*, for 10 min. Afterward, the supernatant was filtered through Chromafil Xtra PA-45/13 nylon filter and injected into the HPLC-RID system. A Polaris Hi-Plex H, 300 × 7.7. column (Agilent Technologies, Santa Clara, CA, USA) was used to separate the compounds having H_2_SO_4_ (5 mM) as a mobile phase, with a flow rate of 0.6 mL/min. The column and RID temperatures were 70 °C and 35 °C, respectively; meanwhile, the compound elution time was 25 min. 

OpenLab—ChemStation software (Agilent Technologies, Santa Clara, CA, USA) was used for data acquisition and result interpretation. The compounds identification was realized through comparison with the standard retention times; meanwhile, the quantification of the compounds was realized using calibration curves.

### 2.5. Fatty Acid Profile

In order to identify the fatty acid methyl esters (FAMEs), a Shimadzu GCMS-QP2010 PLUS (Shimadzu Corporation, Kyoto, Japan) with RT2560 column (100 m × 0.25 mm × 0.20 μm), from the Interdisciplinary Research Platform of Banat University of Agricultural Sciences and Veterinary Medicine King Michael I of Romania was used, according to our previous study, but with some modifications [25].

Briefly, 0.1 g of composite flour or sourdough sample 3 mL of 20% boron trifluoride (BF3) in methanol (Merck KgaA, Darmstadt, Germany) was added and maintained in a water ultrasonic bath at 80 °C (FALC Instruments, Treviglio, Italy) for one hour. 2.5 mL of sodium chloride solution of 10% and 2 mL of hexane were added to each sample, then were centrifuged at 3000 rpm for 15 min, and the FAMEs from the hexane fraction (1 mL) were used for GC-MS analysis. The flow rate of the carrier gas (Helium) was 1 mL·min^−1^, and the splitting ratio was 1:50. The column temperature (100 °C) was maintained for 5 min, and then a gradient at 3 °C/min until 250°C was maintained for 10 min. 

The injection port temperature was 250 °C, and the temperature of the ion source and the GC-MS interface was 210 °C and 255 °C. Fatty acids were identified based on the NIST 05 spectrum library. The fatty acid composition was expressed using the peak area normalization method by relating the peak area corresponding to a given compound to the total area of all peaks. All the analyses were conducted in three replicates. Saturated fatty acids (SFA) were calculated based on the sum of C4:0-C24:0, monounsaturated fatty acids (MUFA) were calculated as the sum of C16:1-C22:1, and polyunsaturated fatty acids (PUFA) were calculated as the sum of C18:2, C18:3, and C20:4. Unsaturated fatty acids (UFA) were calculated as the sum of MUFA and PUFA [26].

### 2.6. Amino Acids Content

A GC (Gas Chromatograph) TRACE™ 1300 (Thermo Scientific, Waltham, CA, USA) was used for the identification and quantification of the amino acids. Briefly, one gram of each sample was grounded and homogenized with 5 mL of distilled water. The obtained samples were centrifuged for 5 min at 2500× *g* and 0.5 mL of the supernatant was passed slowly through a Dowex 50W-W8 exchange resin column and eluted with 4M NH4OH. A two-step derivatization procedure was applied: esterification with butanol-acetyl chloride (4:1 *v*/*v*) for 1 h at 100 °C and trifluoro acetylation with 100 µL trifluoroacetic anhydride at 60 °C for 30 min. 1 µL of the derivatized amino acids were separated on an Rtx-5MS capillary column, 30 m × 0.25 mm, 0.25 µm film thickness, using a temperature program from 50 °C (1 min), increased with 10 °C/min to 100 °C, 4 °C/min to 200 °C and 20 °C/min to 290 °C (hold for 5 min). The injector was kept at 250 °C and the detector at 280 °C. Helium was used as carrier gas with a flow of 1 mL/min.

### 2.7. Qualitative ITEX/GC-MS Volatile Compounds Profile

In-tube extraction technique (ITEX) coupled with a gas chromatograph-mass spectrometer GC-MS QP-2010 (Shimadzu Scientific Instruments, Kyoto, Japan) and a Zebron ZB-5ms capillary column with 0.25 µm thickness were used to extract, separate and quantify the aroma volatile compounds, according to the method described by Chiș et al. [27]. Briefly, 5 g of each sample were introduced in a 20 mL headspace vial, incubated at 60 °C for 20 min under continuous agitation. A porous polymer fiber microtrap (ITEX-2TRAPTXTA, Tenax TA 80/100 mesh, ea) was used to absorb the gaseous phase from the vial, which was further thermally desorbed into the GS-MS injector. Helium was the used carrier gas with a flow rate of 1 mL/min and a split ratio of 1:5. The column parameters were as follows: an initial temperature of 35 °C holds for 5 min, then 170 °C with an increase of 7 °C/min to a final temperature of 260 °C (10 °C/min), for 5 min. The MS detection was performed on a quadrupole mass spectrometer operating in full scan (40–400 *m*/*z*) electron impact (EI) at an ionization energy of 70 eV. NIST27 and NIST147 libraries were used to identify the aroma volatile compounds and compared with www.pherobase.com or www.flavornet.org, (accessed on 10 January 2022) retention drawn indices [28,29]. The results were expressed as a relative percentage of the total peak area.

### 2.8. Minerals Determination through AAS (Atomic Absorption Spectrophotometry)

Macro and microminerals were analyzed through atomic absorption spectrophotometry, as described by Păucean et al. [30] and Chiș et al. [17], using mixed standard solutions (ICP Multi Element Standard solution IV CertiPUR). Shortly, a Nabertherm furnace (Nabertherm B150, Lilienthal, Germany) was used to burn 3 g of each sample for 10 h at a temperature of 550 °C. The obtained samples were recovered with 20% HCl (*w*/*v*) using a volumetric flask of 20 mL and performed through AAS equipment (Varian Spectra 240 FS AA equipment, Varian, Mulgrave, Victoria, Australia). The working conditions were the following: Air: acetylene ratio 13.50:2, Nebulizer uptake rate: 5 L/min.

The detection limits were as follows: Mg (0.02 ppm), K (0.06 ppm), Fe (0.03 ppm), Cu (0.03 ppm), Mn (0.03 ppm), Zn (0.03 ppm). For calibration standard solutions were used with a concentration ranging from 0.3 to 3 μg/L, prepared from a multielement solution ICP Standard solution 1000 mg/L. The working conditions for AAS equipment and minerals wavelength (λ) are presented in Table S1, Supplementary Materials.

### 2.9. Statistical Analysis

All data were analyzed using SPSS 19.0 software (IBM, Portsmouth, UK). ANOVA (one way analysis of variance) at a confidence level of 95% was applied to determine statistically significant differences between the means. When the null hypothesis was rejected, a post hoc comparison test was further performed (Duncan multiple comparison test at *p* < 0.05). Data were analyzed in triplicates and results were expressed as means ± standard deviation.

## 3. Results

### 3.1. Chemical Characterization of Insect Flour

Insect flour proximate composition and minerals compounds are presented in Table 2. Protein highlighted the highest amount; meanwhile, ash reached a value of 5.04%. From the minerals group, K (potassium) exhibited the highest value, followed by P (phosphorus) and Cu (copper). In Table 3, organic acids and alcohols from the insect powder are displayed. Fumaric (13.70 mg/g f.w.) and citric acid (12.87 mg/g f.w.) were the main representatives of the organic acid group. Two alcoholic compounds were identified, which were as follows: 1.3 propandiol (1.3 PD) and 2.3 propandiol (2.3 PD), with values of 1.37 mg/g f.w. and 0.10 mg/g f.w., respectively.

A total number of 10 fatty acids were identified from the insect flour and grouped as follows: SFA (saturated fatty acids), MUFA (monosaturated fatty acids), and PUFA (Polyunsaturated fatty acids). The main fatty acids were linoleic acid, oleic acid, palmitic, and stearic acid, with percentages of 41.91%, 27.27%, 16.29%, and 10.59%, respectively, as shown in Table 4. Aroma volatile compounds were divided into the following groups: alcohols, aldehydes, ketones, terpenes and terpenoids, and others. From the alcohols, 3-methyl-1-butanol was the main compound; meanwhile, hexanal and 2-methyl-5-propan-2-ylcyclohex-2-en-1-one were the main representatives of the aldehydes group, as shown in Table 4.

Regardless of the insect flour amino-acid content, a total number of 24 amino acids, such as ALA (alanine), SAR (sarcosine), Gly (glycine), ABA (Amino-Butyric Acid), VAL (Valine), BaiB (β-aminoisobutyric acid), LEU (leucine), aILE (L-Alloisoleucine), ILE (Isoleucine), THR (Threonine), SER (Serine), PRO (Proline), ASN (Asparagine), TPR (Tetratricopeptide), ASP (Aspartic Acid), MET (Methionine), HYP (Hydroxy Proline), GLU (Glutamic Acid), PHE (Phenylalanine), GPR (G-protein regulatory (GPR) motif), ORN (Ornithine), LYS (Lysine), GLN (Glutamine), and TRP (Tryptophan), were analyzed, from which 16 were quantified. ALA, Gly, aILE, PRO, and Val reached the higher extended values, together with LEU and LYS, as presented in Table 5.

### 3.2. Sourdough Characterization

#### 3.2.1. Microbial growth, pH, TTA (Total Titratable Acidity)

**Figure 1:**
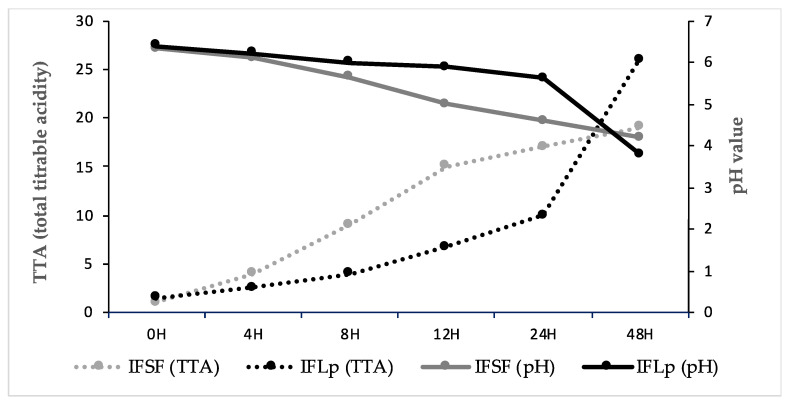
pH and TTA values of fermented sourdough.

The adaptability of the Lp strain was monitored through its microbial growth, pH, and TTA values. The microbial growth for inoculated samples reached the highest value after 24 h of fermentation (9.01 log cfu/mL); meanwhile, at 48 h of fermentation, the value significantly decreased (7.39 log cfu/mL). As presented in Figure 1, the IFLp samples pH reached after 24 h of fermentation a value of 4.6; meanwhile, the spontaneous fermentation, at the same fermentation time, had a value of 5.63. The TTA values increased in both samples with the increased fermentation time, having final values after 24 h of fermentation of 19 and 26 for IFLp and IFSF, respectively.

#### 3.2.2. Sourdough Organic Acids, Fatty Acids, Amino Acids, and Aroma Volatile Compounds

Organic acid content during the fermentation time is displayed in Table 6. Lactic, fumaric, citric and acetic acid were the organic acid compounds identified in the samples. During fermentation, most of them increased their values, reaching the highest values after 24 h of fermentation, mainly in inoculated samples. With respect to fatty acids, controlled fermentation led to an enrichment of the SFA and MUFA groups, whilst PUFA decreased during fermentation (Table 7).

Considering the amino acid evolution of the controlled sourdough fermentation (Table 8), ALA, VAL, LEU, and METH increased their values by 1.76, 3.67, 1.99, and 2.89 hold higher. The same positive trend was also observed in aroma volatile compounds (Table 9), controlled fermentation leading to an enrichment in alcohols such as 3-methyl-1 butanol, 2 methyl-1-butanol, aldehydes (heptanal, hexanal), ketones (2-methyl-5-propan-2-ylcyclohex-2-en-1-one, 2-heptanone), terpens, and tepenoids (from which *p*-Cymene reached the higher extended value after 24 h of fermentation). The spontaneous fermentation led to the formation of volatile compounds such as 1-pentanol, benzoic acid and disulfide-dimethyl, which had as odor perceptions pungent, fermented, bready, wine, faint balsam, and garlic, respectively.

#### 3.2.3. Minerals Sourdough Evolution

Table 10 displays the mineral content of spontaneous and controlled fermentation. Significant differences were emphasized between samples (*p* < 0.05), mainly after 24 h of fermented sourdoughs. Macrominerals such as K, Mg, and Ca reached, after 24 h of controlled fermentation, values of 201.23 mg/100 g, 15.33 mg/100 g, and 1.79 mg/100 g; meanwhile, microminerals such as Cu, Zn, and Mn reached values of 42.03 mg/100 g, 6.52 mg/100 g, and 1.98 mg/100 g, respectively.

## 4. Discussion

*A. domesticus* represents one of the most promising insects in the world, mainly due to its content rich in protein, amino acids, and fat, mainly polyunsaturated fatty acids [31,32]. In the present study, the protein content of the insect flour was 72.60%; meanwhile, Meyer-Rochow et al. [5] reported a protein value for edible insects in a range of 10.6–80.3 g/100 g d.w. In line with this, Williams et al. [33] showed that there was a difference in protein content of *A. domesticus* adult and nymph that varied from 66.6% to 67.2%, respectively. A large body of literature showed that protein insect content could be influenced by their species but also by their developmental stage [5,34]. 

Regardless of the insect mineral composition, recently Atowa et al. [34] showed that there are three main factors that could influence their content such as the differences between species, the food sources of the insects, and seasonal influence. With respect to the mineral content, as reported by Williams et al. [33], the Ca (calcium) content of house crickets could vary in a high range and it is directly correlated with a diet supplemented with Ca. In the present study, Ca reached a value of 1.90 mg/100 g; meanwhile, Williams et al. [33] mentioned a value for *A. domesticus* (nymphs) of 27.5 mg/100 g d.w. The cricket powder Fe content (7.20 mg/100 g) is quite similar to that mentioned by Kosečková et al. [35], who reported an amount between 5.6–6.4 mg/100 g d.w. The difference could be explained by the feed insects and by the type of insect. For instance, it has been reported that Kenya termites could have an Fe amount of 332 mg/100 g d.w. or even 1562 mg/100 g for Kenia cricket species [34]. Moreover, it is important to mention that Fe bioavailability is higher in insect flour than even in beef meat [36]. On the other side, Williams et al. [33] emphasized different values for minerals, such as 352 mg/100 g d.w. for K and 225 mg/100 g d.w. for P, respectively. This could be justified as a result of the insects’ feed source, which is incorporated as the food is being consumed and the food that is already present in the gastrointestinal tract. In line with this, Payne et al. [37] studied the mineral content of 10 different insect species and concluded that the mineral content was very different and could be influenced by feed composition and harvesting season, and also by the geographic location.

The main fatty acids were represented by the PUFAs group, with a value of 43.28% followed by MUFA and SFA, with values of 29.39% and 27.94%, respectively. With respect to fatty acids content, linoleic, oleic, and palmitic acids were mainly identified in the present study. This is in line with, Williams et al. [33] who showed that all insects generally contain linoleic acid, as well as oleic, palmitic, and linolenic fatty acids, but it seems that every insect species has a different pattern with respect to its fatty acid profile. For instance, in the present study, the linoleic acid value (41.91%) was bigger than the amount reported by Messina et al. [38], which was 32.35%; meanwhile, oleic acid (27.47%) was different from that identified by the same authors (33.52%) and could be justified by different stages of development, origin, and species. It is also important to mention one of the main indicators of a healthy diet, which is the ratio between PUFA/SFA. In the present study, the ratio PUFA/MUFA is only 1.47, a value higher than 3 being correlated with different diseases such as different tumors [39].

In the present study, Ala, Gly, aILE, Pro, and Val were the mainly quantified amino acids from the insect flour. With respect to insect flour amino acid content, Atowa et al. [34] identified leucine, tyrosine, valine, and lysine as the main amino acids from the three different insects, while showing that leucine, valine, lysine, and isoleucine [40] were the most important in cricket flour. On the other hand, Boontiam et al. [41] showed that lysine, tyrosine, and leucine were the most common amino acids in crickets (*Gryllus bimaculatus, De Geer,1773*).

Considering the aroma volatile profile of the insect flour, as far as we know, this is the first time when 2-methyl-5-propan-2-ylcyclohex-2-en-1-one is mentioned as being identified in the aldehydes group, with an odor perception of spicy, minty, caraway, bread, and rye bread. Odor perceptions such as fruity, cinnamon, intense green, fruity, aldehydic odor, grass, and leafy are mainly due to volatile compounds such as 2-heptanone and hexanal, respectively.

With respect to insect flour fermentation, parameters such as pH and TTA were first analyzed, being the markers of a good fermentation and acidification rate. The drop in the pH (Figure 1) could be influenced by the organic acid production, leading to an increase in the TTA value [21]. The insect flour sourdough TTA value was quite bigger at the beginning of the fermentation (Figure 1), mainly because of ash insect flour content, which probably generates a higher buffering sourdough capacity [15]. 

For the microorganism growth, carbohydrates are used as a carbon source for microbial energy through carbohydrate metabolic pathways [42]. The degradation of starch and protein leads to substrate enrichment for the LAB growth and is responsible for carbohydrates’ fermentation into end products such as organic acids [21]. 

In the present study, organic acids such as lactic, acetic, and succinic increased their values mainly through controlled fermentation (Table 6). This could be justified by the *L. plantarum* strain, which is claimed by the literature as having an exceptionally broad capacity in different phytochemicals’ metabolic conversion through enzymes such as reductases, decarboxylases, glycosyl hydrolases, or even phenolic acid esterases [20]. The production of weak organic acids such as lactic and acetic ones through fermentation with heterofermentative LAB is claimed by a large body of literature—[19,26,43,44], —and it is supported by a low pH value, caused by LAB acidification matrix through fermentation [43]. Generally, acetic and lactic acids are characterized by Nissen et al. [14] as performance fermentation process indicators for every LAB inoculum being the main metabolites of heterofermentative lactobacilli with a positive impact on the sensorial quality and safety of the final fermented food. 

An important role in the TCA (tricarboxylic acid) cycle also entitled Krebs or citric acid cycle is played by citric, fumaric, succinic, and malic acids [41]. Key enzymes of citric acid metabolism such as citrate permease or citrate lyase enhance the formation of succinic acid; meanwhile, citrate lyase results in decarboxylation to pyruvate that can be further converted to α -acetolactate, which could be enzymatically reduced to 3-hydroxybutan-2-one. Citrate conversion to succinic acid is more common in *Lactobacillus* strains [38] and probably, could justify the increase during fermentation of succinic acid in the present study. On the other hand, the production of succinic acid through LAB fermentation is also mentioned by Wang et al. [45], who highlighted that some LAB strains are able to use citrate transporters and generate succinic acid by using fumaric and malic acids. 

The decrease of citric acid during fermentation could be explained by its consumption by LAB, mainly, when they are low in carbohydrates [46]. In line with this, our research group highlighted that citric acid could be used by *Lactobacillus plantarum* strains as an energy supply, decreasing its content through fermentation [17]. Moreover, citric acid is claimed by the literature as having a positive effect on textural and sensorial bread characteristics, mainly on its crumb-softening aftermath and flavor, respectively [17,46].

Fumaric acid is considered as an antibacterial agent mainly used as a beverage ingredient and food acidulant, being 1.5-fold more acidic than citric acid [47]. It is considered a natural organic acid and an intermediate in the citric acid cycle [48] and, to some extent, it seems that amino acids and fatty acid content could also enhance its production [47].

An increase in fatty acids during fermentation was mainly observed in the controlled fermentation, values being significantly different from the spontaneous one (Table 7). The increase in fatty acids content during fermentation was also debated by (Castro-López et al. [49] and Ogawa et al. [50], who mentioned that lactic acid bacteria could perform different fatty acids transformations through isomerization, hydration, dehydration, and saturation. Moreover, Hayek and Ibrahim [51] explained that some LABs could have intracellular or extracellular enzymes, such as lipases that could be involved in the breakdown of lipids into fatty acids and glycerol. Furthermore, Rasi et al. [52] emphasized a close relationship between the *Lactobacillus* genus and the lipids metabolism. In line with this, the formation of aroma volatile compounds such as aldehydes (hexanal, heptanal, octanal), ketones and alcohols could be also a result of lipid oxidation by lipoxygenase enzymes, as shown by Petel et al. [53]. Moreover, recently, Maiyo et al. [54] showed that the use of cricket (*Scapsipedus icipe* Hugel and Tanga) powder in porridge products leads to an enrichment in fatty acid amount and fermentation enhanced a significant PUFA content increase.

With respect to the amino acids’ evolution during controlled fermentation, a positive trend can be observed, mainly in the fermentation with Lp, at 24 h. This could be justified by the capacity of Lp to metabolize different food chemical compounds, mainly protein, which ends with the formation of amino acids and peptides [26]. The results obtained in the present study are consistent with those of Mendoza-Salazar et al. [55] who mentioned that fermentation of grasshopper sauces with *Aspergillus oryzae* strain leads to an enhancement of the amino acids content.

Regarding the aroma volatile compounds, the controlled fermentation led to the formation of compounds such as 3-methyl-1-butanol, with odor perceptions of whiskey, malt, and burnt 2-methyl-5-propan-2-ylcyclohex-2-en-1-one, with odor perceptions of spicy, minty, caraway, bread, and rye bread; meanwhile, the spontaneous fermentation enhanced the formation of benzoic acid and disulfide dimethyl, with a faint and unpleasant odor perception. The sourdough dynamic transformation of volatile derivatives in controlled fermentation is explained by amino acid reactions such as transamination, deamination, decarboxylation, and side chain modifications, which end with the development of alcohols, aldehydes, and acids [26]. In line with this, the development of benzaldehyde could be a result of metabolic degradation of phenylalanine, whilst, 3-methyl-1-butanol is considered one of the most frequently identified compounds and could derive from the leucine degradation [56].

The increased mineral values through fermentation with Lp strain are consistent with earlier works of our research group, such as [17,24]. This could be explained by the drop in the pH value, which leads to the activation of the phytase Lp activity. The phytate reduction amount through fermentation with *Lactobacillus plantarum* was also emphasized by Sharma et al. [57].

Moreover, it was stated that phytate and tannin, which were identified in cricket edible insects [5], are able to hinder or to inhibit mineral absorption especially. Fermentation could be used as a tool to decrease their value, leading to a better mineral bioavailability [17]. It seems that the anti-nutritional content is correlated mainly with the plant’s chemical composition, which insects feed upon, but also to the environment and to the growing plant conditions [5]. 

## 5. Conclusions

The *Lactobacillus plantarum* ATCC 8014 strain highlighted a good adaptability during insect flour fermentation, leading to a sourdough enriched in bioactive compounds such as fatty acids, amino-acids, and minerals. Moreover, the dynamics of aroma volatile compounds showed that controlled fermentation was conducive to the formation of aldehydes, ketones, terpens, and terpenoids, with pleasant odor perception such as benzaldehyde, 2-methyl-5-propan-2-ylcyclohex-2-en-1-one, *p*-Cymene and β-Myrcene. To successfully explore the nutritional characteristics of the obtained insect sourdough fermented with *Lactobacillus plantarum* ATCC 8014 strain, further studies should use this in the development of new products.

## Figures and Tables

**Table 1:** Sampling codes.

Sample Codes		
Sampling Times (h)	Sourdough Spontaneous Fermentation (IFSF)	Sourdough with *Lactobacillus plantarum* Fermentation (IFLp)
0	IFSF0H	IFLp0H
4	IFSF4H	IFLp4H
8	IFSF8H	IFLp8H
12	IFSF12H	IFLp12H
24	IFSF24H	IFLp24H
48	IFSF48H	IFLp48H

**Table 2:** Chemical composition and mineral content of insect flour (IF).

Parameters	Insect Flour
Protein (%) *	72.60 ± 0.34
Fat (%) *	6.13 ± 0.22
Ash (%) *	5.04 ± 0.33
Moisture (%) *	6.67 ± 0.67
Minerals (mg/100 g d.w.)	
K	560.46 ± 0.94
P	150.80 ± 0.85
Cu	46.16 ± 0.24
Zn	15.11 ± 0.77
Mg	11.32 ± 0.44
Fe	7.20 ± 0.32
Mn	2.21 ± 0.11
Ca	1.90 ± 0.22
Ni	0.14 ± 0.04

* fresh weight; d.w.—dry weight.

**Table 3:** Insect flour (IF) organic acids content.

Parameters	IF	Retention Times (min)
Organic acids (mg/g f.w.)		
Oxalic acid	1.70 ± 0.05	7.78
Citric acid	12.87 ± 0.07	9.45
Succinic acid	5.71 ± 0.05	12.88
Lactic acid	8.51 ± 0.07	13.77
Fumaric acid	13.70 ± 0.21	14.63
Acetic acid	0.55 ± 0.06	15.83
Ascorbic acid	1.04 ± 0.04	10.50
Alcohols (mg/g f.w.)		
1.3 PD	1.37 ± 0.03	18.01
2.3 PD	0.10 ± 0.05	18.88

IF—insect flour; n.d.—not detected, f.w.—fresh weight.

**Table 4:** Fatty acid content and volatile profile of insect flour.

Shorthand Nomenclature	Fatty Acid Name	Type	IF (%) *	
14:0	Myristic	SFA	0.51 ± 0.01	
16:0	Palmitic	SFA	16.29 ± 0.18	
C16:1 cis	Palmitoleic	MUFA	0.41 ± 0.03	
C16:1 ω − 7	Palmitolinoleic	MUFA	0.73 ± 0.02	
17:0	Margaric acid	SFA	0.21 ± 0.03	
18:0	Stearic acid	SFA	10.59 ± 0.21	
18:1 (n − 9)	Oleic acid	MUFA	27.47 ± 0.07	
18:2 (n − 6)	Linoleic acid	PUFA, ω − 6	41.91 ± 0.04	
20:0	Arachidic	SFA	0.33 ± 0.02	
18:2 (n − 3)	Linolenic	PUFA, ω − 3	1.37 ± 0.03	
∑ SFA			27.94 ± 0.45	
∑ MUFA			29.39 ± 0.14	
∑ PUFA			43.28 ± 0.07	
∑ PUFAs/SFAs			1.47 ± 0.15	
**Volatile profile**				
Volatile compounds			Characteristic odor	Conc. (% of the total peak area) *
*Alcohols*				
1-Pentanol			Pungent, fermented, bready, wine.	n.d.
3-methyl-1-Butanol			Whiskey, malt, burnt	8.55 ± 0.22
2-methyl-1-Butanol			Malt	7.99 ± 0.31
*Aldehydes*				
Hexanal			Intense green, fruity, aldehydic odor, grass, leafy	17.93 ± 0.46
Heptanal			Fresh, Aldehydic, Fatty, Green.	n.d.
Benzaldehyde			Almond, fruity, powdery, nutty	6.34 ± 0.55
2,4-Nonadienal, (E,E)-			Fatty, waxy odor	2.33 ± 0.19
Octanal			fruit-like odor	n.d.
*Ketones*				
Acetophenone			Floral, Almond	3.18 ±0.03
2-methyl-5-propan-2-ylcyclohex-2-en-1-one			Spicy, minty, caraway, bread, rye bread	16.00 ± 0.18
2-Heptanone			Fruity, cinnamon	14.56 ± 0.88
*Terpenes and terpenoids*				
*p*-Cymene			Citrus, Sweet, Herbal, Spicy	5.52 ± 0.77
β-Myrcene			Balsamic, must, spice	10.65 ± 0.22
Benzoic Acid			Faint, Balsam	1.22 ± 0.33
*Others*				
Dimethyl-disulfide			Garlic	5.75 ± 0.09

n.d.—not detected, * f.w.—fresh weight.

**Table 5:** Insect flour amino-acid content.

Amino Acids (mg/g f.w.)	
*Essential amino acid*	
Leucine	45.00 ± 0.08
Lysine	46.14 ± 0.12
Phenylalanine	18.58 ± 0.33
Isoleucine	23.70 ± 0.21
Methionine	11.77 ± 0.55
Threonine	14.86 ± 0.42
Tryptophan	18.48 ± 0.78
Valine	41.53 ± 0.83
Total	220.06 ± 3.32
*Non-essential amino acid*	
Alanine	221.21 ± 0.88
Glycine	93.73 ± 0.66
Amino-Butyric Acid	n.d.
β-aminoisobutyric acid	n.d.
L-Alloisoleucine	88.28 ± 0.34
Serine	7.56 ± 0.55
Sarcosine	n.d.
Proline	84.26 ± 0.39
Asparagine	n.d.
Tetratricopeptide	n.d.
Aspartic Acid	16.77 ± 0.52
Hydroxy Proline	n.d.
Glutamic Acid	37.72 ± 0.49
G-protein regulatory (GPR) motif	n.d.
Glutamine	n.d.
Ornithine	10.74 ± 0.77
Total	560.27 ± 4.60

n.d.—not detected; f.w.—fresh weight.

**Table 6:** Organic acid evolution during insect flour fermentation.

Samples	Oxalic Acid mg/100 g	Citric Acid mg/100 g	Ascorbic Acid mg/100 g	Succinic Acid mg/100 g	Lactic Acid mg/100 g	Fumaric Acid mg/100 g	Acetic Acid mg/100 g	1.3 PD mg/100 g	2.3 BD mg/100 g
IFSF0H	0.34 ± 0.02 ^a^	3.04 ± 0.05 ^c^	0.26 ± 0.04 ^a^	1.48 ± 0.03 ^bc^	2.11 ± 0.05 ^b^	3.58 ± 0.07 ^de^	0.14 ± 0.03 ^a^	0.34 ± 0.02 ^a^	0.04 ± 0.01 ^a^
IF*Lp*0H	0.36 ± 0.02 ^a^	3.10 ± 0.21 ^c^	0.24 ± 0.11 ^a^	1.35 ± 0.04 ^ab^	2.01 ± 0.05 ^b^	3.43 ± 0.03 ^d^	0.16 ± 0.02 ^a^	0.33 ± 0.02 ^a^	0.05 ± 0.01 ^a^
IFSF4H	0.39 ± 0.03 ^a^	2.99 ± 0.02 ^c^	0.21 ± 0.03 ^a^	1.41 ± 0.02 ^abc^	2.03 ± 0.03 ^b^	3.23 ± 0.08 ^cd^	0.12 ± 0.03 ^a^	0.30 ±0.04 ^a^	0.03± 0.01 ^a^
IF*Lp*4H	0.38 ± 0.02 ^a^	3.01 ± 0.03 ^c^	0.27 ± 0.02 ^a^	1.31 ± 0.04 ^a^	2.12 ± 0.03 ^b^	3.14 ± 0.22 ^cd^	0.18 ± 0.01 ^a^	0.28 ± 0.05 ^a^	0.04 ± 0.02 ^a^
IFSF8H	0.37 ± 0.02 ^a^	3.27 ± 0.03 ^cd^	0.26 ± 0.01 ^a^	1.35 ± 0.02 ^ab^	1.88 ± 0.03 ^b^	3.20 ± 0.22 ^cd^	0.34 ± 0.11 ^ab^	0.36 ± 0.02 ^a^	0.03 ± 0.01 ^a^
IF*Lp*8H	0.57 ± 0.02 ^b^	3.65 ± 0.05 ^de^	0.30 ± 0.02 ^a^	1.53 ± 0.03 ^cd^	2.57 ± 0.05 ^bc^	2.90 ± 0.04 ^bc^	0.21 ± 0.03 ^a^	0.40 ± 0.07 ^a^	0.04 ± 0.02 ^a^
IFSF12H	0.64 ± 0.02 ^bc^	3.04 ± 0.03 ^c^	0.21 ± 0.02 ^a^	1.68 ± 0.22 ^d^	2.01 ± 0.21 ^b^	3.40 ± 0.11 ^d^	0.57 ± 0.12 ^b^	0.45 ± 0.03 ^a^	0.06 ± 0.03 ^a^
IF*Lp*12H	0.57 ± 0.03 ^b^	3.92 ± 0.02 ^e^	0.32 ± 0.03 ^ab^	1.90 ± 0.05 ^de^	3.02 ± 0.07 ^c^	2.36 ± 0.06 ^a^	0.87 ± 0.02 ^c^	0.43 ± 0.03 ^a^	0.06 ± 0.02 ^a^
IFSF24H	0.64 ± 0.03 ^bc^	2.50 ± 0.05 ^b^	0.27 ± 0.03 ^a^	1.69 ± 0.06 ^d^	2.25 ± 0.04 ^b^	3.56 ± 0.05 ^de^	0.93 ± 0.02 ^c^	1.09 ± 0.04 ^b^	0.12 ± 0.03 ^ab^
IF*Lp*24H	0.83 ± 0.05 ^d^	3.07 ± 0.04 ^c^	0.38 ± 0.03 ^b^	2.24 ± 0.05 ^f^	3.62 ± 0.05 ^d^	2.51 ± 0.03 ^ab^	1.27 ± 0.03 ^d^	0.42 ± 0.05 ^a^	0.06 ± 0.02 ^a^
IFSF48H	0.81 ± 0.02 ^d^	2.01 ± 0.02 ^a^	n.d.	1.86 ± 0.05 ^de^	0.63 ± 0.03 ^a^	4.01 ± 0.02 ^e^	2.03 ± 0.04 ^e^	0.90 ± 0.03 ^b^	0.33 ± 0.05 ^c^
IF*Lp*48H	0.91 ± 0.03 ^d^	2.88 ± 0.03 ^bc^	n.d.	2.14 ± 0.03 ^f^	2.57 ± 0.02 ^bc^	3.14 ± 0.05 ^cd^	1.89 ± 0.06 ^e^	0.39 ± 0.04 ^a^	0.21 ± 0.02 ^b^

Different small letters in a column indicate significant difference (*p* < 0.05) for the same compound between samples at different moments of fermentation (0, 4, 8, 12, 24, and 48 h); each value was the mean of triplicate measurements; n.d.—not detected, f.w.—fresh weight.

**Table 7:** Sourdough fatty acids evolution during fermentation (% for total compounds).

Shorthand Nomenclature	Fatty Acid Name	Type	IFSF0H	IF*Lp*0H	IFSF4H	IF*Lp*4H	IFSF8H	IF*Lp*8H	IFSF12H	IF*Lp*12H	IFSF24H	IF*Lp*24H	IFSF48H	IF*Lp*48H
14:0	Myristic	SFA	0.45 ± 0.02 ^a^	0.43 ± 0.02 ^a^	0.48 ± 0.03 ^a^	0.38 ± 0.02 ^a^	0.49± 0.03 ^a^	0.45 ± 0.02 ^a^	0.48 ± 0.02 ^a^	1.62 ± 0.07 ^b^	0.66 ± 0.09 ^a^	1.55 ± 0.02 ^b^	0.69 ± 0.03^a^	1.78 ± 0.05^b^
16:0	Palmitic	SFA	14.08 ± 0.03 ^a^	14.11 ± 0.04 ^a^	15.03 ± 0.02 ^a^	15.99 ± 0.18 ^ab^	14.22 ± 0.05 ^a^	17.99 ± 0.63 ^b^	24.56 ± 0.77 ^c^	26.85 ± 0.39 ^d^	26.07 ± 0.84 ^cd^	29.04 ± 0.55 ^e^	27.62 ± 0.77^d^	30.22 ± 0.55^e^
C16:1 cis	Palmitoleic	MUFA	0.22 ± 0.03 ^a^	0.24 ± 0.03 ^ab^	0.22 ± 0.01 ^a^	0.24 ± 0.03 ^ab^	0.21 ± 0.02 ^a^	0.33 ± 0.01 ^b^	0.30 ± 0.01 ^b^	0.37 ± 0.03 ^b^	0.20 ± 0.01 ^a^	0.40± 0.06 ^bc^	0.22 ± 0.03^a^	0.22± 0.05 ^a^
C16:1 ω - 7	Palmitolinoleic	MUFA	0.40 ± 0.02 ^a^	0.43 ± 0.02 ^a^	0.41 ± 0.04 ^a^	0.40 ± 0.02 ^a^	0.43 ± 0.05 ^a^	0.61 ± 0.05 ^b^	0.67 ± 0.04 ^b^	0.71 ± 0.05 ^b^	0.67 ± 0.03 ^b^	0.40 ± 0.03 ^a^	0.41 ± 0.02 ^a^	0.44 ± 0.01 ^a^
17:0	Margaric acid	SFA	0.15 ± 0.05 ^a^	0.18 ± 0.03 ^ab^	0.12 ± 0.02 ^a^	0.22 ± 0.01 ^ab^	0.20 ± 0.03 ^ab^	0.27 ± 0.03 ^b^	0.21 ± 0.02 ^ab^	0.27 ± 0.02 ^b^	0.21 ± 0.02 ^ab^	0.17± 0.03 ^a^	0.18 ± 0.05^ab^	0.18 ± 0.02 ^ab^
18:0	Stearic acid	SFA	9.83 ± 0.04 ^b^	10.11 ± 0.02 ^b^	10.00 ± 0.05 ^b^	9.99 ± 0.44 ^b^	9.89 ± 0.39 ^b^	10.00 ± 0.33 ^b^	9.32 ± 0.06 ^a^	10.26 ± 0.53 ^b^	9.43 ± 0.19 ^a^	10.81 ± 0.09 ^c^	10.79 ± 0.03^c^	10.56± 0.55 ^c^
18:1 (n - 9)	Oleic acid	MUFA	18.99 ± 0.02 ^a^	19.01 ± 0.04 ^a^	19.06 ± 0.12 ^a^	19.0 ± 0.23 ^a^	20.08 ± 0.46 ^ab^	20.99 ± 0.29 ^bc^	21.28 ± 0.55 c	22.66 ± 0.88 ^d^	23.00 ± 0.36 ^d^	25.45 ± 0.55 ^e^	26.99 ± 0.72^f^	28.09 ± 0.09
18:2 (n -6 )	Linoleic acid	PUFA, ω - 6	33.77 ± 0.03 ^f^	34.02 ± 0.07 ^f^	33.88 ± 0.33 ^f^	33.99 ± 0.47 ^f^	32.52 ± 0.88 ^ef^	34.77 ± 0.55 ^f^	23.00 ± 0.44 ^a^	24.66 ± 0.44 ^ab^	27.55 ± 0.68 ^c^	30.05 ± 0.44 ^d^	25.81± 0.88^b^	27.77 ± 0.03^c^
20:0	Arachidic	SFA	n.d.	n.d.	n.d.	n.d.	n.d.	n.d.	n.d.	n.d.	n.d.	n.d.	n.d.	n.d.
18:2 (n - 3)	Linolenic	PUFA, ω - 3	0.52 ± 0.05 ^a^	0.52 ± 0.03 ^a^	0.52 ± 0.03 ^a^	0.77± 0.04 ^ab^	0.99 ± 0.05 ^ab^	0.88 ± 0.07 ^ab^	1.02 ± 0.04 ^b^	1.94 ± 0.05 ^d^	1.04 ± 0.05 ^b^	1.40 ± 0.07 ^c^	0.49 ± 0.07^a^	0.90 ± 0.05^ab^
∑ SFA			24.52 ± 0.09^a^	24.84± 0.06 ^a^	25.64± 0.12 ^a^	26.59 ± 0.65 ^b^	24.81 ± 0.93 ^a^	28.72 ± 1.01 ^c^	34.59 ± 0.85 ^d^	39.01 ± 1.01 ^e^	36.39 ± 1.14 ^d^	41.58 ± 0.69 ^f^	39.31± 0.92 ^e^	42.74 ± 1.17 ^f^
∑ MUFA			19.62 ± 0.06^a^	19.68± 0.06 ^a^	19.69± 0.15 ^a^	19.64 ± 0.28 ^a^	20.72 ± 0.06 ^b^	21.93 ± 0.61 ^c^	22.26 ± 0.60 ^c^	23.75 ± 1.30 ^d^	23.88 ± 0.40 ^d^	26.26 ± 0.64 ^e^	27.62 ± 0.77 ^f^	29.56 ± 0.12 ^g^
∑ PUFA			34.30 ± 0.08^e^	34.55± 0.10 ^e^	34.41 ± 0.36 ^e^	34.76 ± 0.51 ^e^	33.51 ± 0.93 ^e^	35.65 ± 0.62 ^f^	24.02 ± 0.48 ^a^	26.61 ± 0.49 ^b^	28.59 ± 0.73 ^c^	31.45 ± 0.51 ^d^	26.30 ± 0.95 ^b^	28.67 ± 0.08 ^c^
∑ PUFAs/SFAs			1.40	1.39	1.34	1.31	1.35	1.24	0.69	0.68	0.79	0.76	0.67	0.67

Different small letters in a column indicate significant difference (*p* < 0.05) for the same compound between samples at different moments of fermentation (0, 4, 8, 12, 24, and 48 h); each value was the mean of triplicate measurements; n.d.—not detected.

**Table 8:** Sourdough amino acids evolution (mg/g f.w.).

Amino acids	IFSF0H	IF*Lp*0H	IFSF4H	IF*Lp*4H	IFSF8H	IF*Lp*8H	IFSF12H	IFLp12H	IFSF24H	IFLp24H	IFSF48H	IF*Lp*48H
*Essential amino acid*												
Leucine	25.09 ± 0.05 ^bc^	25.21 ± 0.55 ^bc^	25.11 ± 0.55 ^bc^	24.89 ± 0.33 ^bc^	26.88 ± 0.88 ^c^	29.99 ± 0.55 ^d^	21.09 ± 0.65 ^a^	39.66 ± 0.75 ^e^	22.55 ± 0.22 ^ab^	49.99 ± 0.77 ^f^	24.00 ± 0.77 ^bc^	50.33 ± 0.72 ^f^
Lysine	21.09 ± 0.88 ^a^	21.55 ± 0.77 ^a^	21.67 ± 0.88 ^a^	21.98 ± 0.83 ^ab^	22.09 ± 0.41 ^ab^	23.99 ± 0.99 ^bc^	24.62 ± 0.63 ^c^	30.67 ± 0.29 ^e^	25.88 ± 0.44 ^c^	39.00 ± 0.44 ^g^	27.98 ± 0.54 ^d^	37.37 ± 0.42 ^f^
Phenylalanine	9.23 ± 0.22 ^a^	9.45 ± 0.29 ^a^	9.31 ± 0.36 ^a^	9.56 ± 0.77 ^a^	9.67 ± 0.99 ^a^	10.88 ± 0.88 ^b^	11.02 ± 0.34 ^b^	15.00 ± 0.39 ^d^	12.88 ± 0.32 ^c^	37.09 ± 0.27 ^e^	13.99 ± 0.38 ^d^	39.03 ±0.53 ^f^
Isoleucine	11.09 ± 0.88 ^a^	11.21 ± 0.44 ^a^	11.44 ± 0.11 ^a^	11.88 ± 0.77 ^a^	12.03 ± 0.11 ^a^	14.01 ± 0.55 ^b^	12.77 ± 0.71 ^ab^	18.74 ± 0.59 ^c^	14.09 ± 0.82 ^b^	28.99 ± 0.37 ^e^	21.66 ± 0.41 ^d^	30.00 ± 0.64 ^ef^
Methionine	5.25 ± 0.22 ^b^	5.30 ± 0.11 ^b^	5.27 ± 0.67 ^b^	5.40 ± 0.87 ^b^	5.88 ± 0.22 ^b^	6.39 ± 0.57 ^b^	3.12 ± 0.31 ^a^	12.77 ± 0.48 ^c^	5.13 ± 0.93 ^b^	15.22 ± 0.36 ^d^	5.99 ± 0.81 ^b^	16.04 ± 0.67 ^d^
Threonine	7.22 ± 0.32 ^a^	7.44 ± 0.21 ^a^	7.33 ± 0.22 ^a^	7.55 ± 0.38 ^a^	7.09 ± 0.44 ^a^	7.99 ± 0.23 ^ab^	8.09 ± 0.42 ^ab^	10.22 ± 0.62 ^b^	9.11 ± 0.39 ^ab^	15.66 ± 0.55 ^d^	9.22 ± 0.39 ^ab^	13.35 ± 0.42 ^c^
Tryptophan	8.66 ± 0.31 ^a^	8.69 ± 0.55 ^a^	8.90 ± 0.88 ^a^	9.03 ± 0.77 ^a^	9.21 ± 0.67 ^a^	9.55 ± 0.62 ^ab^	9.33 ± 0.50 ^a^	11.50 ± 0.55 ^c^	9.90 ± 0.61 ^ab^	12.55 ± 0.62 ^d^	10.22 ± 0.31 ^b^	13.03 ± 0.52 ^d^
Valine	22.04 ± 0.02 ^a^	22.33 ± 0.22 ^a^	22.08 ± 0.66 ^a^	22.33 ± 0.29 ^a^	23.04 ± 0.33 ^a^	28.6 ± 0.77 ^b^	22.89 ± 0.29 ^a^	49.06 ± 0.61 ^c^	26.09 ±0.31 ^b^	80.99 ± 0.41 ^e^	27.77 ± 0.42 ^b^	75.55 ± 0.93 ^d^
Total	109.67 ^a^	111.18 ^ab^	111.11 ^ab^	112.62 ^b^	115.89 ^c^	131.40 ^e^	112.93 ^b^	187.62 ^g^	125.63 ^d^	279.49 ^i^	140.83 ^f^	274.7 ^h^
*Non-essential amino acid*												
Alanine	119.09 ± 0.03 ^a^	119.55 ± 0.79 ^a^	120.04 ± 0.99 ^a^	120.33 ± 0.33 ^a^	120.99 ± 0.44 ^ab^	125.99 ± 0.66 ^c^	121.93 ± 0.81 ^ab^	199.03 ± 0.55 ^d^	123.09 ± 0.77 ^b^	210.03 ± 0.83 ^e^	127.03 ± 0.28 ^c^	201.54 ± 0.08 ^d^
Glycine	54.58 ± 0.05 ^a^	55.02 ± 0.99 ^a^	55.03 ± 0.33 ^a^	55.03 ± 0.99 ^a^	54.09 ± 0.77 ^a^	60.22 ± 0.88 ^b^	54.58 ± 0.73 ^a^	73.60 ± 0.22 ^c^	56.09 ± 0.88 ^a^	76.88 ± 0.65 ^d^	111.82 ± 0.53 ^d^	77.99 ± 0.87 ^e^
Amino-Butyric Acid	n.d.	n.d.	n.d.	n.d.	n.d.	n.d.	n.d.	n.d.	n.d.	n.d.	n.d.	n.d.
β -aminoisobutyric acid	n.d.	n.d.	n.d.	n.d.	n.d.	n.d.	n.d.	n.d.	n.d.	n.d.	n.d.	n.d.
L-Alloisoleucine	40.23 ± 0.27 ^a^	40.55 ± 0.22 ^a^	40.12 ± 0.88 ^a^	40.33 ± 0.88 ^a^	41.09 ± 0.22 ^a^	43.09 ± 0.88 ^b^	42.00 ± 0.83 ^ab^	51.00 ± 0.53 ^d^	43.00 ± 0.44 ^b^	66.00 ± 0.15 ^f^	44.00 ± 0.51 ^c^	63.00 ± 0.52 ^e^
Serine	3.33 ± 0.12 ^ab^	3.64 ± 0.11 ^ab^	3.89 ± 0.11 ^ab^	3.99 ± 0.21 ^ab^	3.99 ± 0.55 ^ab^	4.55 ± 0.71 ^b^	2.77 ± 0.83 ^a^	7.56 ± 0.25 ^c^	3.09 ± 0.22 ^ab^	17.09 ± 0.48 ^d^	5.99 ± 0.55 ^bc^	18.09 ± 0.33 ^d^
Proline	40.22 ± 0.87 ^a^	40.56 ± 0.22 ^a^	41.03 ± 0.99 ^a^	40.89 ±0.22 ^a^	40.33 ± 0.34 ^a^	42.04 ± 0.99 ^b^	43.09 ± 0.76 ^bc^	64.44 ± 0.11 ^e^	44.09 ± 0.36 ^cd^	78.99 ± 0.52 ^f^	45.66 ± 0.36 ^d^	80.44 ± 0.98 ^f^
Asparagine	n.d.	n.d.	n.d.	n.d.	n.d.	n.d.	n.d.	n.d.	n.d.	n.d.	n.d.	n.d.
Tetratricopeptide	n.d.	n.d.	n.d.	n.d.	n.d.	n.d.	n.d.	n.d.	n.d.	n.d.	n.d.	n.d.
Aspartic Acid	7.44 ± 0.45 ^a^	6.99 ± 0.33 ^a^	7.55 ± 0.22 ^a^	7.04 ± 0.33 ^a^	7.44 ± 0.33 ^a^	8.09 ± 0.31 ^ab^	8.00 ± 0.67 ^ab^	14.03 ± 0.25 ^c^	8.66 ± 0.45 ^ab^	16.83 ± 0.88 ^d^	9.05 ± 0.65 ^b^	17.09 ± 0.82 ^d^
Hydroxy Proline	n.d.	n.d.	n.d.	n.d.	n.d.	n.d.	n.d.	n.d.	n.d.	n.d.	n.d.	n.d.
Glutamic Acid	25.73 ± 0.89 ^a^	25.94 ± 0.88 ^a^	25.66 ± 0.99 ^a^	25.88 ± 0.44 ^a^	25.71 ± 0.67 ^a^	26.78 ± 0.65 ^a^	26.83 ± 0.52 ^a^	33.02 ± 0.71 ^c^	27.04 ± 0.67 ^a^	40.23 ± 0.65 ^d^	33.71 ± 0.49 ^c^	28.77 ± 0.88 ^b^
G-protein regulatory (GPR) motif	n.d.	n.d.	n.d.	n.d.	n.d.	n.d.	n.d.	n.d.	n.d.	n.d.	n.d.	n.d.
Ornithine	3.78 ± 0.34 ^a^	3.99 ± 0.12 ^a^	4.03 ± 0.17 ^a^	4.34 ± 0.32 ^ab^	4.77 ± 0.33 ^ab^	7.11 ± 0.44 ^c^	5.75 ± 0.60 ^b^	9.20 ± 0.33 ^d^	6.25 ± 0.58 ^bc^	11.09 ± 0.39 ^e^	6.77 ± 0.67 ^c^	11.88 ± 0.55 ^e^
Total	294.40 ^a^	296.24 ^ab^	297.35 ^b^	297.83 ^b^	298.41 ^b^	317.87 ^e^	304.95 ^c^	451.88 ^g^	311.31 ^d^	517.13 i	384.03 ^f^	498.80 ^h^

Different small letters in a row indicate significant difference (*p* < 0.05) for the same compound between samples at different moments of fermentation (0, 4, 8, 12, 24, and 48 h); each value was the mean of triplicate measurements; f.w.—fresh weight; n.d.—not detected.

**Table 9:** Volatilome insect sourdough profile (% of the total peak area).

Volatile Compounds	Perceived Flavor	IFSF0H	IF*Lp*0H	IFSF4H	IF*Lp*4H	IFSF8H	IF*Lp*8H	IFSF12H	IF*Lp*12H	IFSF24H	IF*Lp*24H	IFSF48H	IF*Lp*48H
**Alcohols**													
1-Pentanol	Pungent, fermented, bready, wine	n.d.	n.d.	0.58 ± 0.02 ^a^	0.31± 0.03 ^a^	1.77 ± 0.02 ^b^	0.22± 0.01 ^a^	2.02 ± 0.03 ^bc^	0.16 ± 0.02 ^a^	2.62 ± 0.02 ^c^	0.23 ± 0.03 ^a^	6.62 ± 0.03 ^d^	1.77 ± 0.03 ^b^
3-methyl-1-butanol	Whiskey, malt, burnt	7.01 ± 0.03 ^a^	6.99 ± 0.06 ^a^	7.22 ± 0.03 ^a^	7.33 ± 0.22 ^a^	7.38 ± 0.07 ^a^	18.09 ± 0.05 ^d^	15.67 ± 0.05 ^c^	19.57 ± 0.08 ^e^	18.89 ± 0.05 ^d^	21.77 ± 0.05 f	16.33 ± 0.11 ^cd^	10.02 ± 0.05 ^b^
2-methyl-1-butanol	malt	5.55 ± 0.02 ^a^	5.67 ± 0.08 ^a^	5.77 ± 0.05 ^a^	6.33 ± 0.33 ^a^	9.88 ± 0.44 ^b^	10.99 ± 0.21 ^bc^	12.55 ± 0.03 ^c^	12.09 ± 0.05 ^c^	13.93 ± 0.03 ^d^	14.22 ± 0.05 ^d^	10.22 ± 0.77 ^b^	5.99 ± 0.03 ^a^
*Total*		12.56 ± 0.05 ^a^	12.66 ± 0.12 ^a^	13.57± 0.55 ^ab^	13.97 ± 0.58 ^b^	19.03 ± 0.53 ^d^	29.30 ± 0.27 ^e^	30.24 ± 0.11 ^e^	31.82 ± 0.15 ^f^	35.44 ± 0.1 ^h^	36.22 ± 0.12 ^i^	33.17± 0.91 ^g^	17.78 ± 0.11 ^c^
**Aldehydes**													
Hexanal	Intense green, fruity, aldehydic odor, grass, leafy	17.64 ± 0.07 ^d^	17.06 ± 0.09 ^d^	15.03 ± 0.55 ^c^	16.22 ± 0.24 ^d^	14.55 ± 0.98 ^c^	15.09 ± 0.89 ^c^	16.89 ± 0.33 ^d^	18.23 ± 0.09 ^e^	18.15 ± 0.06 ^e^	4.21 ± 0.06 ^a^	13.22 ± 0.67 ^b^	20.31 ± 0.07 ^f^
Heptanal	Fresh, Aldehydic, Fatty, Green.	1.33 ± 0.02 ^a^	1.19 ± 0.03 ^a^	1.51 ± 0.33 ^a^	1.56 ± 0.88 ^a^	1.77 ± 0.05 ^a^	2.75 ± 0.08 ^b^	1.89 ± 0.22 ^ab^	3.89 ± 0.14 ^c^	2.68 ± 0.01 ^b^	5.32± 0.02 ^d^	2.88 ± 0.23 ^b^	5.03 ± 0.23 ^d^
Benzaldehyde	Almond, fruity, powdery, nutty	3.28 ± 0.03 ^b^	3.23 ± 0.02 ^b^	3.40 ± 0.03 ^bc^	3.29 ± 0.03 ^b^	3.11 ± 0.03 ^b^	3.55 ± 0.11 ^bc^	3.01 ± 0.16 ^b^	3.89 ± 0.22 ^c^	1.80 ± 0.05 ^a^	6.12 ± 0.07 ^d^	2.45 ± 0.05 ^ab^	4.02 ± 0.12 ^c^
2,4-Nonadienal, (E,E)-	Fatty, waxy odor	5.99 ± 0.05 ^c^	6.77 ± 0.07 ^d^	6.84 ± 0.04 ^d^	7.03 ± 0.29 ^d^	7.89 ± 0.22 ^e^	5.73 ± 0.33 ^c^	8.88 ± 0.71 ^f^	0.44 ± 0.05 ^a^	6.04 ± 0.22 ^c^	0.12 ± 0.05 ^a^	3.86 ± 0.80 ^b^	5.95 ± 0.34 ^c^
Octanal	Fruit-like odor	0.29 ± 0.01 ^ab^	0.32 ± 0.05 ^ab^	0.33 ± 0.06 ^ab^	0.21 ± 0.05 ^ab^	0.99 ± 0.11 ^bc^	0.45 ± 0.05 ^ab^	2.77 ± 0.44 ^d^	0.66 ± 0.09 ^ab^	0.27 ± 0.55 ^ab^	0.88 ± 0.03 ^b^	0.02 ± 0.01 ^a^	1.35 ± 0.13 ^c^
*Total*		28.53 ± 0.18 ^d^	28.57 ± 0.26 ^d^	27.11 ± 1.01 ^c^	28.31 ± 1.49 ^d^	28.31 ± 1.39 ^d^	27.57 ± 0.1.46 ^cd^	33.44 ± 1.86 ^e^	27.11 ± 0.59 ^c^	28.94 ± 0.89 ^d^	16.65 ± 0.23 ^a^	22.42 ± 1.76 ^b^	36.66 ± 0.61 ^f^
**Ketones**													
Acetophenone	Floral, Almond	1.25 ± 0.03 ^ab^	1.21 ± 0.05 ^ab^	1.01 ± 0.22 ^a^	1.22 ± 0.56 ^ab^	1.54 ± 0.04 ^b^	1.79 ± 0.88 ^b^	1.41 ± 0.05 ^ab^	2.50 ± 0.08 ^c^	1.66 ± 0.07 ^b^	3.22± 0.11 d	1.99 ± 0.06 ^bc^	1.56 ± 0.12 ^b^
2-methyl-5-propan-2-ylcyclohex-2-en-1-one	Spicy, minty, caraway, bread rye bread	7.77 ± 0.06 ^bc^	7.99 ± 0.11 ^b^	7.11 ± 0.08 ^b^	7.15 ± 0.38 ^b^	8.20 ± 0.03 ^bc^	9.66 ± 0.09 ^d^	8.50 ± 0.32 ^c^	11.06 ± 0.03 ^e^	4.47 ± 0.11 ^a^	12.49± 0.05 ^f^	8.68 ± 0.33 ^c^	11.04 ± 0.05 ^e^
2-Heptanone	Fruity, cinnamon	20.09 ± 0.03 ^f^	19.55 ± 0.22 ^f^	23.25 ± 0.14 ^g^	23.44 ± 0.88 ^g^	16.25 ± 0.72 ^d^	15.22 ± 0.07 ^c^	12.33 ± 0.08 ^a^	15.74 ± 0.05 ^cd^	13.11 ± 0.05 ^ab^	17.73 ± 0.02 ^e^	14.11 ± 0.55 ^b^	18.31 ± 0.88 ^e^
*Total*		29.11 ± 0.12 ^e^	28.75 ± 0.38 ^e^	31.35 ± 0.24 ^g^	31.81 ± 1.82 ^g^	25.99 ± 0.14 ^d^	26.67 ± 1.04 ^d^	22.24 ± 0.45 ^b^	29.30 ± 0.16 ^e^	19.24 ± 0.23 ^a^	33.44 ± 0.17 ^h^	24.78 ± 0.94 ^c^	30.91 ± 2.45 ^f^
**Terpenes and terpenoids**													
*p*-Cymene	Citrus, Sweet, Herbal, Spicy	3.20 ± 0.07 ^bc^	3.11± 0.02 ^bc^	3.09± 0.05 ^bc^	3.65 ± 0.03 ^bc^	3.22 ± 0.03 ^bc^	4.09 ± 0.02 ^c^	2.88 ± 0.02 ^b^	5.55 ± 0.21 ^d^	2.55 ± 0.03 ^b^	6.99 ± 0.07 ^e^	1.05 ± 0.22 ^a^	3.89 ± 0.11 bc
β-Myrcene	Balsamic, must, spice	5.53 ± 0.03 ^d^	5.12 ± 0.03 ^d^	4.55 ± 0.03 ^c^	5.00 ± 0.02 ^d^	4.01 ± 0.04 ^bc^	5.50 ± 0.05 ^d^	3.55 ± 0.03 ^bc^	5.53 ± 0.05 ^d^	3.01 ± 0.02 ^b^	6.49 ± 0.05 ^e^	2.01 ± 0.05 ^a^	5.00 ± 0.04 ^d^
*Total*		8.73 ± 0.10 ^d^	8.23 ± 0.05 ^d^	7.64 ± 0.08 ^c^	8.65 ± 0.05 ^d^	7.23 ± 0.07 ^c^	9.59 ± 0.07 ^e^	6.43± 0.05 ^bc^	11.08 ± 0.26 ^f^	5.56 ± 0.05 ^b^	13.48 ± 0.12 ^g^	3.06 ± 0.27 ^a^	8.89 ± 0.05 ^d^
**Acids**													
Benzoic Acid	Faint, balsam	2.09 ± 0.02 ^b^	2.71 ± 0.05 ^bc^	2.55 ± 0.11 ^b^	1.50 ± 0.10 ^b^	3.09 ± 0.55 ^c^	0.67 ± 0.03 ^a^	03.03 ± 0.04 ^c^	0.19 ± 0.02 ^a^	5.09 ± 0.07 ^d^	0.12 ± 0.02 ^a^	9.58 ± 0.04 ^e^	0.55 ± 0.03 ^a^
total		2.09 ± 0.02 ^b^	2.71 ±0.05 ^bc^	2.55 ± 0.11 ^b^	1.50 ± 0.10 ^b^	3.09 ± 0.76 ^c^	3.09 ± 0.03 ^a^	0.67 ± 0.04 ^c^	0.19 ± 0.02 ^a^	5.09 ± 0.07 ^d^	0.12 ± 0.02 ^a^	9.58 ± 0.04 ^e^	0.55 ± 0.03 ^a^
**Others**													
Disulfide, dimethyl	Garlic	4.35 ± 0.05 ^d^	4.29 ± 0.04 ^d^	3.22 ± 0.05 ^c^	3.11 ± 0.88 ^c^	3.77 ± 0.21 ^cd^	2.05 ± 0.03 ^b^	5.22 ± 0.11 ^e^	0.50 ± 0.04 ^a^	5.78 ± 0.10 ^e^	0.11 ± 0.11 ^a^	7.03 ± 0.43 ^f^	5.31 ± 0.88 ^e^
*Total*		4.35 ± 0.05 ^d^	4.29 ± 0.04 ^d^	3.22 ± 0.05 ^c^	3.11 ± 0.88 ^c^	3.77 ± 0.21 ^cd^	2.05 ± 0.03 ^b^	5.22 ± 0.11 ^e^	0.50 ± 0.04 ^a^	5.78 ± 0.10 ^e^	0.11 ± 0.11 ^a^	7.03 ± 0.43 ^f^	5.31 ± 0.88 ^e^

Different small letters in a row indicate significant difference (*p* < 0.05) for the same compound between samples at different moments of fermentation (0, 4, 8, 12, 24 and 48 h); each value was the mean of triplicate measurements; n.d.—not detected.

**Table 10:** Insect flour mineral evolution during fermentation.

Samples	Ca	Mg	K	P	Cu	Cr	Ni	Zn	Fe	Mn
	mg/100 g	mg/100 g	mg/100 g	mg/100 g	mg/100 g	mg/100 g	mg/100 g	mg/100 g	mg/100 g	mg/100 g
IFSF0H	0.64 ± 0.03 ^a^	10.31 ± 0.59 ^a^	179.73 ± 0.88 ^a^	67.36 ± 0.17 ^ab^	31.90 ± 0.39 ^a^	0.26 ± 0.03 ^a^	0.13 ± 0.05 ^a^	4.67 ± 0.12 ^abc^	4.32± 0.05 ^abc^	0.82 ± 0.88 ^a^
IF*Lp*0H	0.66 ± 0.05 ^a^	10.47 ± 0.88 ^a^	180.09 ± 0.73 ^a^	68.03 ± 0.59 ^ab^	32.23 ± 0.88 ^a^	0.30 ± 0.02 ^a^	0.20 ± 0.03 ^ab^	4.88 ± 0.22 ^bc^	4.34 ± 0.03 ^abc^	0.91 ± 0.36 ^a^
IFSF4H	0.66 ± 0.02 ^a^	10.50 ± 0.76 ^a^	180.03 ± 0.92	68.55 ± 0.93 ^ab^	32.03 ± 0.54 ^a^	0.29 ± 0.08 ^a^	0.27± 0.04 ^ab^	4.55 ± 0.13 ^abc^	4.55 ± 0.11 ^bc^	0.88 ± 0.37 ^a^
IF*Lp*4H	0.69 ± 0.07 ^a^	11.01 ± 0.91 ^ab^	180.09 ± 0.13 ^a^	68.02 ± 0.69 ^ab^	33.01 ± 0.71 ^abc^	0.33 ± 0.02 ^a^	0.25 ± 0.07 ^ab^	4.99 ± 0.03 ^cd^	4.45 ± 0.25 ^bc^	1.02 ± 0.06 ^ab^
IFSF8H	0.70 ± 0.03 ^a^	10.66 ± 0.05 ^ab^	183.99 ± 0.99 ^b^	68.09± 0.53 ^ab^	32.55 ± 0.93 ^a^	0.30 ± 0.05 ^a^	0.19 ± 0.05 ^ab^	4.03 ± 0.06 ^ab^	4.03 ± 0.19 ^ab^	0.91 ± 0.08 ^a^
IF*Lp*8H	0.91 ± 0.06 ^a^	11.89 ± 0.04 ^b^	187.02 ± 0.83 b	70.23 ± 0.58 ^b^	36.02 ± 0.71 ^d^	0.67 ± 0.02 ^a^	0.57 ± 0.09 ^b^	5.22 ± 0.03 ^cd^	4.99 ± 0.08 ^cd^	1.23 ± 0.09 ^b^
IFSF12H	0.73 ± 0.04 ^a^	10.90 ± 0.03 ^ab^	185.82 ± 0.71 ^b^	67.34 ± 0.53 ^ab^	31.55 ± 0.94 ^a^	0.15 ± 0.03 ^a^	0.15 ± 0.03 ^a^	4.00 ± 0.05 ^ab^	3.88 ± 0.17 ^ab^	1.03 ± 0.05 ^ab^
IF*Lp*12H	1.22 ± 0.08 ^b^	13.09 ± 0.27 ^c^	193.03 ± 0.74 ^c^	73.05 ± 0.98 ^c^	39.27 ± 0.73 ^e^	1.20 ± 0.07 ^b^	1.01 ± 0.07 ^c^	5.86 ± 0.03 ^de^	5.58 ± 0.26 ^de^	1.55 ± 0.03 ^c^
IFSF24H	0.81 ± 0.05 ^a^	11.00 ± 0.52 ^ab^	186.09 ± 0.91 ^b^	66.55 ± 0.82 ^a^	32.99 ± 0.83 ^ab^	0.21 ± 0.05 ^a^	0.25 ± 0.09 ^ab^	3.89 ± 0.02 ^a^	3.55 ± 0.21 ^a^	1.22 ± 0.07 ^b^
IF*Lp*24H	1.79 ± 0.09 ^c^	15.33 ± 0.18 ^d^	201.23 ± 0.83 ^d^	78.12 ± 0.93 ^d^	42.03 ± 0.94 ^f^	1.70 ± 0.03 ^c^	1.33 ± 0.06 ^cd^	6.52 ± 0.21 ^e^	6.05 ± 0.87 ^e^	1.98 ± 0.06 ^d^
IFSF48H	0.84 ± 0.10 ^a^	10.89 ± 0.32 ^ab^	185.77± 0.91 ^b^	65.99 ± 0.83 ^a^	33.05 ± 0.72 ^abc^	0.25 ± 0.02 ^a^	0.29 ± 0.03 ^ab^	4.01 ± 0.03 ^ab^	3.72 ± 0.09 ^ab^	1.44 ± 0.03 ^c^
IF*Lp48*H	1.85 ± 0.08 ^c^	15.37 ± 0.33 ^d^	200.88 ± 0.88 ^d^	79.01 ± 0.59 ^d^	41.88 ± 0.86 ^f^	1.78 ± 0.04 ^c^	1.42 ± 0.05 ^cd^	6.77 ± 0.35 ^e^	6.29 ± 0.18 e	2.02 ± 0.07 ^d^

Different small letters in a column indicate significant difference (*p* < 0.05) for the same compound between samples at different moments of fermentation (0, 4, 8, 12, 24 and 48 h); each value was the mean of triplicate measurements; d.w.—dry weight.

## Data Availability

The data presented in this study are available on request from the corresponding author.

## Supplementary Materials

The following supporting information can be downloaded at: https://www.mdpi.com/article/10.3390/insects13070576/s1, Table S1: Working conditions for Varian Spectra 240 FS spectrophotometer.
