# Supplementary material for: The Impact of Insect Flour on Sourdough Fermentation-Fatty Acids, Amino-Acids, Minerals and Volatile Profile
Source: Insects. 2022 Jun 24;13(7):576. doi: 10.3390/insects13070576 (PMC9322958; doi:10.3390/insects13070576)
Supplement: Supplementary file 1 [file insects-13-00576-s001.zip › insects-1738917-supplementary.pdf]

Table S1. Working conditions for Varian Spectra 240 FS spectrophotometer

Metal	λ (nm)	Lamp current (mA)	Slit width
Ni	232	4	0.2
Cr	357.9	7	0.2
Cu	324.8	4	0.5
Cd	228.8	4	0.5
Mn	279.5	5	0.2
Zn	213	5	1
Fe	248.3	5	0.2
